# Copy Number Variation: What Is It and What Has It Told Us About Child Psychiatric Disorders?

**DOI:** 10.1016/j.jaac.2013.05.013

**Published:** 2013-08

**Authors:** Anita Thapar, Miriam Cooper

**Affiliations:** Institute of Psychological Medicine and Clinical Neurosciences and the Medical Research Council (MRC) Centre for Neuropsychiatric Genetics and Genomics at Cardiff University School of Medicine

Copy number variation is now recognized as an important class of risk factor for several child psychiatric disorders. In this article, we first explain what copy number variants (CNVs) are. We then consider key findings and what these have told us about the etiology of these conditions. Finally, we discuss whether these findings can yet translate into clinical practice.

## What Are Copy Number Variants?

There are multiple types of genetic alterations that contribute to human variability, many of which have yet to be fully characterized or properly understood. This genetic variation can involve differences in the DNA nucleotide sequence as well as changes in chromosome structure. Single nucleotide polymorphisms (SNPs) are common variants of individual nucleotide sequence that are frequently observed in the population (>1%). CNVs are a type of structural variant involving alterations in the number of copies of specific regions of DNA, which can either be deleted or duplicated. These chromosomal deletions and duplications involve fairly large stretches of DNA (that is, thousands of nucleotides [>1 kb], which may span many different genes) but can range considerably in size as well as prevalence. As is the case for other types of genetic mutations, some CNVs are inherited whereas others spontaneously arise de novo. To date, most CNV research in psychiatry has focused on rare forms of copy number variation that occur with a frequency of <1%.

## What Are the CNV Findings for Child Psychiatric Disorders?

There are several well-characterized rare developmental phenotypes caused by CNVs of known pathogenicity, such as Velocardiofacial, Prader-Willi, and Smith-Magenis syndromes. Although the role of most CNVs is far less clear, there is now growing evidence that the genetic architecture of more common psychiatric and neurodevelopmental conditions includes different types of both common and rare genetic variation.^1^ An increased burden of rare CNVs has been observed and replicated in several conditions. These include autism spectrum disorder (ASD), attention-deficit/hyperactivity disorder (ADHD), and intellectual disability (ID), as well as schizophrenia. CNVs also contribute to risk of idiopathic epilepsy.

Risk factors for complex disorders tend to be of small effect size (odds ratios of <2), and this has certainly proved to be the case for individual SNPs. Conversely, the types of CNVs that have been found to be associated with neurodevelopmental and psychiatric disorders have been of considerably greater effect size.^2^, ^3^ This is not surprising, because as the types of CNV mutations that have been studied are so rare, their effects have to be large to be detected in modest sample sizes. In fact, so far large rare CNVs show the strongest and most consistent associations, and size and burden of CNVs seem to be proportional to phenotypic severity. Large rare CNVs appear to be further enriched in individuals who have ASD or ADHD with comorbid ID,^3^, ^4^ and the burden appears to be even greater in those with ID and congenital anomalies.^2^, ^3^

Psychiatric disorders mainly have a complex, multifactorial etiology. Apart from some infrequent exceptions (e.g., known genetic syndromes, fetal alcohol syndrome, congenital rubella), a single risk, whether genetic or environmental, is neither necessary nor sufficient to result in disorder. Thus, typical of all complex disease epidemiology, many carriers of risk CNVs will not display a psychiatric or neurodevelopmental phenotype, and not all those affected will possess the risk variant. As an analogy, although cigarette smoking is a well-established risk factor of large effect size, not all smokers develop lung cancer, and not all individuals with lung cancer have smoked. There are likely multiple types of risks and pathways that lead to the same child psychiatric disorder.

## What Have CNV Findings Told Us?

### CNV Mutations Can Arise De Novo

Genetic data from parents are required to identify whether variants are inherited or have arisen de novo. So far, although CNVs have been found to be both inherited and de novo in origin, much interest has focused on de novo mutations. Such mutations are associated with risk of ASD, ID, and schizophrenia but would not contribute to the familial transmission of these disorders. Increased paternal age has been found to be associated with some types of mutations, as well as with risks of both ASD and schizophrenia. Rare de novo CNVs do not, however, necessarily explain all the observed paternal age effect.

### CNVs Span Traditional Diagnostic Boundaries

Although ASD, ADHD, schizophrenia, and ID have clinically distinctive defining features, one key and consistent finding is that the CNVs associated with each of these disorders show significant overlap with each other.^1^, ^5^ That is, the same CNV is associated with increased risk of different types of disorders: this is known as pleiotropy. This phenomenon is most consistently observed for disorders considered to have strong neurodevelopmental origins (ASD, ADHD, schizophrenia, and ID). This observation highlights that the same genetic risks operate across diagnostic categories. That is also true for other risk factors.

### CNVs Can Provide Clues to Biology

The chromosomal regions that harbor CNVs observed to be associated with psychiatric disorder are located at multiple genomic regions on many different chromosomes, and each deletion or duplication often encompasses several genes. So, are these simply randomly distributed mutations, or are their locations significant? Multiple types of evidence suggest that CNV locations provide meaningful clues to the neurobiology of these disorders. Genes code for proteins that are involved in different biological processes. The functions of different genetic elements are not fully understood; however, genetic findings can be used to make inferences about the biological processes involved. Although there are some methodological limitations to this approach, consistent patterns of findings are emerging. The rare CNVs found to be associated with ASD, ADHD, schizophrenia, and ID all seem to span genes that converge on meaningful biological processes. For example, CNV studies of ASD, schizophrenia, and ID have strongly implicated disrupted synaptic function. Studies of ADHD have suggested involvement of nicotinic acetylcholine receptor pathways, glutamatergic transmission (also found in schizophrenia), and genes involved in neural development.

## What Are the Clinical Implications—and What Next?

To interpret CNV findings in a clinically meaningful way is challenging. Essentially it is difficult to prove causal pathogenic effects. Moreover, most child psychiatric disorders, including those that have been found to be associated with CNVs, are multifactorial in origin and not caused by a single risk factor. In many cases, the presence of a specific CNV will not necessarily imply that it played a role in causing disorder, and, for some CNVs, carriers can be unaffected. Inferring causality is further complicated by the high levels of comorbidity between different child psychiatric and developmental disorders. Furthermore, incomplete penetrance, pleiotropic risk effects, and de novo mutations make estimating the risk of disorder recurrence particularly complex. The etiological role of environmental factors and gene–environment interplay in complex disorders should also not be overlooked—nor should the contribution of other classes of genetic variant.

Advances in techniques for detecting submicroscopic chromosomal abnormalities have meant screening for rare CNVs in idiopathic ID has become increasingly commonplace. Rare, syndromic forms of ASD and other psychiatric disorders will likely require similar types of assessment and counseling. However, for most children who are under the care of child psychiatrists, it seems unlikely that routine CNV testing will become established practice in the near future for the majority of individuals unless perhaps they also have ID or multiple developmental morbidities. Prevention and treatment strategies based directly on genetics also remain distant.

Nonetheless, the establishment of CNVs as risks for psychiatric disorders is an important breakthrough. The findings highlight commonalities across different neurodevelopmental disorders. The genes indexed by CNVs are beginning to provide valuable biological insights and clues to patterns of altered brain development and function that accompany disorders such as ASD, ADHD, and schizophrenia. CNV findings, by shedding light on pathogenesis, can contribute to progress in developing novel interventions and risk modification strategies that target relevant risk processes in the nearer future.

## References

[1] Sullivan P.F., Daly M.J., O'Donovan M. (2012). Genetic architectures of psychiatric disorders: the emerging picture and its implications. Nat Rev Genet.

[2] Cooper G.M., Coe B.P., Girirajan S. (2011). A copy number variation morbidity map of developmental delay. Nat Genet.

[3] Girirajan S., Brkanac Z., Coe B.P. (2011). Relative burden of large CNVs on a range of neurodevelopmental phenotypes. PLoS Genet.

[4] Williams N.M., Zaharieva I., Martin A. (2010). Rare chromosomal deletions and duplications in attention-deficit hyperactivity disorder: a genome-wide analysis. Lancet.

[5] Williams N.M., Franke B., Mick E. (2012). Genome-wide analysis of copy number variants in attention deficit hyperactivity disorder: the role of rare variants and duplications at 15q13.3. Am J Psychiatry.

